# Digital twins as global learning health and disease models for preventive and personalized medicine

**DOI:** 10.1186/s13073-025-01435-7

**Published:** 2025-02-07

**Authors:** Xinxiu Li, Joseph Loscalzo, A. K. M. Firoj Mahmud, Dina Mansour Aly, Andrey Rzhetsky, Marinka Zitnik, Mikael Benson

**Affiliations:** 1 Medical Digital Twin Research Group, Department of Clinical Sciences Intervention and Technology, Karolinska Institute, Stockholm, Sweden; 2 Brigham and Women’s Hospital, Harvard Medical School, Boston, USA; 3 Department of Medical Biochemistry and Microbiology, Uppsala University, 75105 Uppsala, Sweden; 4 Departments of Medicine and Human Genetics, Institute for Genomics and Systems Biology, University of Chicago, Chicago, USA; 5 Department of Biomedical Informatics, Harvard Medical School, Cambridge, MA USA; 6 Broad Institute of MIT and Harvard, Cambridge, MA USA; 7 Kempner Institute for the Study of Natural and Artificial Intelligence, Harvard Data Science Initiative, Harvard University, Cambridge, MA USA

**Keywords:** Digital twins, Personalized medicine, Data integration, Computational methods

## Abstract

Ineffective medication is a major healthcare problem causing significant patient suffering and economic costs. This issue stems from the complex nature of diseases, which involve altered interactions among thousands of genes across multiple cell types and organs. Disease progression can vary between patients and over time, influenced by genetic and environmental factors. To address this challenge, digital twins have emerged as a promising approach, which have led to international initiatives aiming at clinical implementations. Digital twins are virtual representations of health and disease processes that can integrate real-time data and simulations to predict, prevent, and personalize treatments. Early clinical applications of DTs have shown potential in areas like artificial organs, cancer, cardiology, and hospital workflow optimization. However, widespread implementation faces several challenges: (1) characterizing dynamic molecular changes across multiple biological scales; (2) developing computational methods to integrate data into DTs; (3) prioritizing disease mechanisms and therapeutic targets; (4) creating interoperable DT systems that can learn from each other; (5) designing user-friendly interfaces for patients and clinicians; (6) scaling DT technology globally for equitable healthcare access; (7) addressing ethical, regulatory, and financial considerations. Overcoming these hurdles could pave the way for more predictive, preventive, and personalized medicine, potentially transforming healthcare delivery and improving patient outcomes.

## Background

Ineffective medication is one of the most important healthcare problems. Many patients with complex diseases do not respond to treatment or experience serious side effects. This problem causes enormous suffering and costs for health care, drug development, and production loss. An important reason for ineffective medication is the daunting complexity of diseases. Multi-omics analyses down to the single cell level show that each disease can involve altered interactions among thousands of genes across billions of cells in multiple organs [1].

Most diseases, including inflammatory, cardiovascular, malignant, and metabolic, can develop for many years, or even decades, before symptoms manifest themselves and a diagnosis is given. Ineffective treatment increases the risk of comorbidities, and a vicious circle of increasing treatment inefficiency ensues.

Disease progression can differ between different patients with the same diagnosis or within a patient at different time points. Indeed, health and disease can be seen as variable entities on continuous scales. Such variations depend on genetic or environmental factors, such as pollution, lifestyle, and inequitable health care. The 2030 agenda for sustainable development identified effective and equitable health as priorities [2]. To address these priorities would require identification of factors that predispose to, or protect against, a complex disease in the life of a patient.

Digital twins (DTs) can contribute to these goals. The DT concept is derived from engineering with the aims of modeling and developing complex systems more effectively and inexpensively in silico than in real life. As with many emerging disciplines, there is no accepted definition of a medical DT [3]. However, many definitions have been proposed, ranging from a computational model of a disease process or a comprehensive model of a whole virtual representation of a patient that is continuously updated with relevant information [4].

Reasons for lack of a generally accepted definition include the wide variety of potential applications of medical DTs and emerging technologies. Thus, it is possible that definitions will change, and perhaps be adapted to different contexts. This flexibility was also proposed in a recent publication about medical DTs [3].

Here, we will use a broad definition of medical DTs: virtual representations of healthy or sick processes across lifecycles that can be understood, learned, and reasoned with real-time data or simulation models to predict, prevent, or treat diseases [5].

Early examples of DTs have already been tested in the clinic, such as in the setting of an artificial lung or artificial pancreas [6–8]. Recently a resource of sex-specific, organ-resolved whole-body models (WBMs) of infant metabolism was described [9]. This can be used to develop personalized infant-WBMs to predict infant growth in health and disease. Similar models of the whole immune system are projected [10].

Ideally, analyses and computational treatment of DTs will improve health care by paving the way for predictive, preventive, and personalized treatments [11–13]. Two recent literature reviews provide comprehensive compilations of potential DT applications in health care [14, 15], as summarized in Table 1.

**Table 1:** Examples of digital twin applications in health care and organizations to promote such applications

Applications	Examples
Organ models	Artificial lungs and artificial pancreas [6–8]
System models	Infant metabolism, immune system [10]
Disease models	Cancer, cardiology, neurology, orthopedics, and wellness [11, 16–35]
Drug discovery	Improve drug discovery [36, 37]
Clinical trial design	Improve clinical trial design [9, 38]
Hospital workflows	Optimize hospital processes and provide clinical decision support for treatment of individual patients [39, 40]
**Organizations to promote such applications**	
European Virtual Human Twin	Aims at development, integration, and adoption of patient-specific predictive computer models, for clinical decision support system [41]
US National Science Foundation (NSF), NIH and FDA	Awarded more than $6 M across 7 new projects aiming at digital twins for therapeutic and clinical use [42]
Chinese Medical Association	Promotes and disseminates knowledge about medical digital twins in China [43]
Digital Twins for Health Consortium	20 institutions, focusing on developing digital twin infrastructure for lung cancer, sepsis, mental health, diabetes, leukemia, and cardiovascular diseases [44]
Swedish Digital Twin Consortium	Universities and hospitals aiming to predict and prevent disease trajectories [45]
Siemens	Industrial models of the heart and for pharmaceutical development [46, 47]

DTs have been applied in cancer, cardiology, neurology, orthopedics, and wellness [11, 16–35, 48]. Other applications include the use of DTs to improve drug discovery, clinical trial design, and workflows in hospitals [9, 36–40].

As an example, Siemens Healthineers and the Medical University of South Carolina collaborated to optimize hospital processes based on DT applications that simulated different workflows and medical equipment [40].

Another example was a DT of a hospital that provided predictive models of health care needs during the COVID-19 pandemic. Those needs included ventilators, critical care beds, and extracorporeal life support. The generated DTs were used to optimize the use of such resources and to provide clinical decision support for treatment of individual patients in all hospitals in the state of Oregon [39].

The medical potential of DTs has been recognized by scientific organizations in the US, Europe, and Asia, and has led to international collaborative efforts to implement this computational strategy in health care and clinical trials. Such efforts and potential clinical applications have been extensively reviewed [6, 48–60].

However, clinical implementation of DTs involves multiple challenges that have not been systematically addressed in the same review, including (1) dynamic characterization of health and disease-associated molecular changes on population-, organome-, cellulome-, and genome-wide scales, as well as environmental factors; (2) computational methods that integrate and organize all changes into DT; (3) prioritization of mechanisms, from which (4) diagnostic biomarkers and preventive measures or therapeutic targets can be inferred; (5) solutions to connect 1–4 so that DTs can learn from each other; (6) user-friendly interfaces adapted to individuals and care givers; (7) solutions to disseminate DTs on a global scale for equitable and effective health; and (8) solutions to address social, psychological, organizational, ethical, regulatory, and financial challenges and opportunities. As highlighted by manifestos about DTs from the European Commission and US National Academy of Sciences, Engineering and Medicine, there is a lack of concrete clinical implementations that address these challenges [55, 56].

Moreover, the emerging market for medical DTs is projected to reach US$183 billion by 2031 [61]. This has resulted in multiple industrial efforts to develop and implement DTs for health care [62].

Here, we will discuss these challenges and potential solutions and give concrete examples of such solutions.

## 1. Dynamic and multi-scale characterization of health and risk factors

**Fig. 1:**
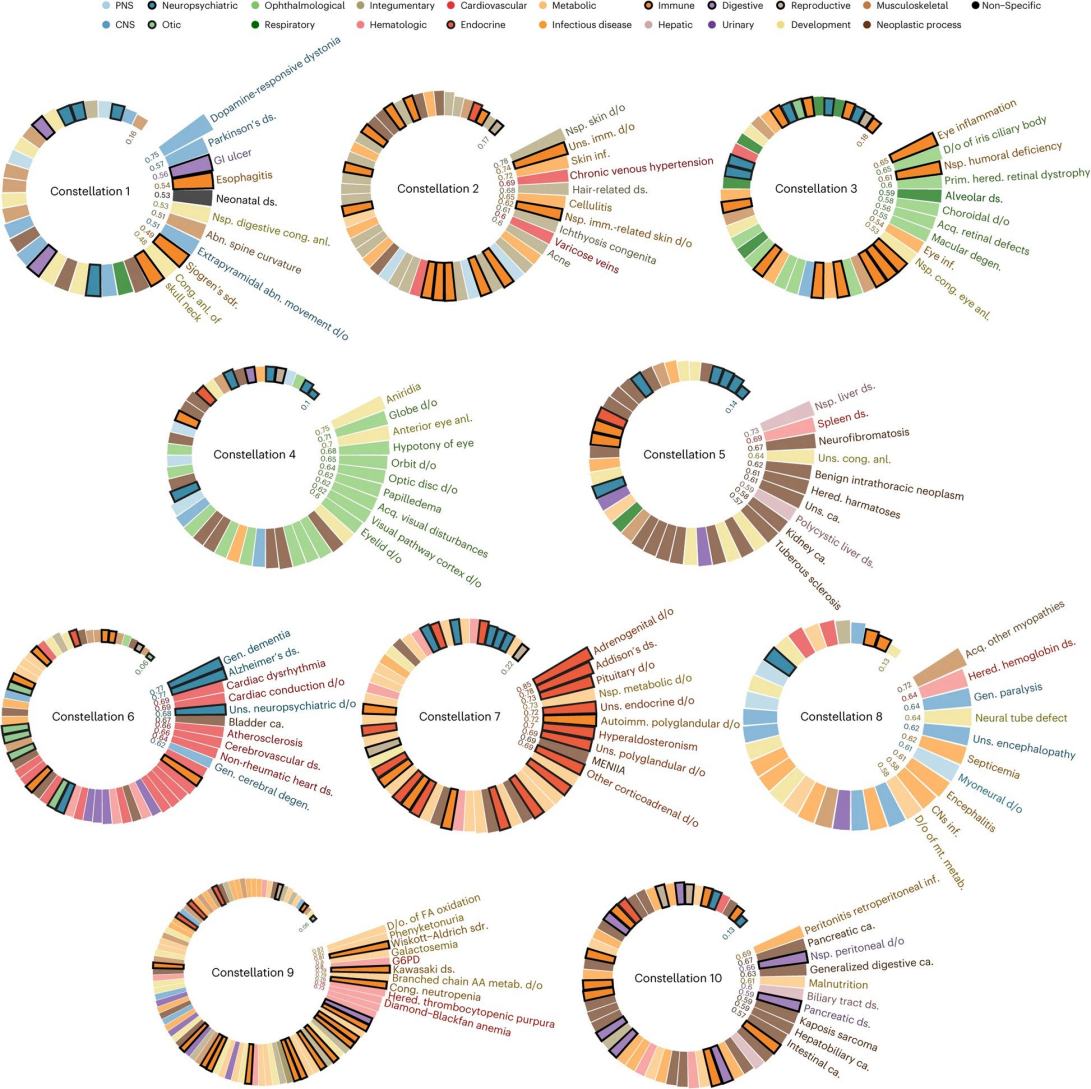
Disease complexity and heterogeneity in populations and individuals, figure from [63]. Disease constellations in a population identified by analyses of a longitudinal diagnostic registry of 200 million Americans

Predictive, preventive, and personalized medicine will require analyses of potential disease causes on multiple scales ranging from populations to individuals, to their tissues, cells, and molecular species. Since multi-morbidity is common, population-wide analyses are important for characterizing disease constellations. This goal is feasible because of the availability of longitudinal electronic medical records of populations and large biobanks. As an example, see our analyses of temporal disease trajectories of over 200 million Americans revealing ten constellations of comorbid diseases (Fig. 1).

The motivations for studying whole populations include that environmental and genetic factors associated with health and disease may be identified. As an example, a study of health records of over 480,000 (United States) US individuals, along with geographically specific environmental quality measures, suggested that different combinations of genetic and environmental factors play significant roles in schizophrenia risk. The authors concluded that such knowledge would have the potential to implement preventative public health measures at the level of the general population, as well as personalized clinical strategies through genotype-guided primary, secondary, and tertiary prevention to protect defined individuals from exposure to specific environmental risks [64]. Moreover, diseases often occur sequentially, so that disease trajectories can be characterized. Such information might be used for prediction and prevention of diseases. A well-known example from health care today is that early diagnosis and treatment of hypertension prevent cardiovascular diseases. However, many disease trajectories and their genetic/environmental associations may remain uncharacterized because of their complexity and heterogeneity, as well as lack of systematic analyses on population-wide scales.

**Fig. 2:**
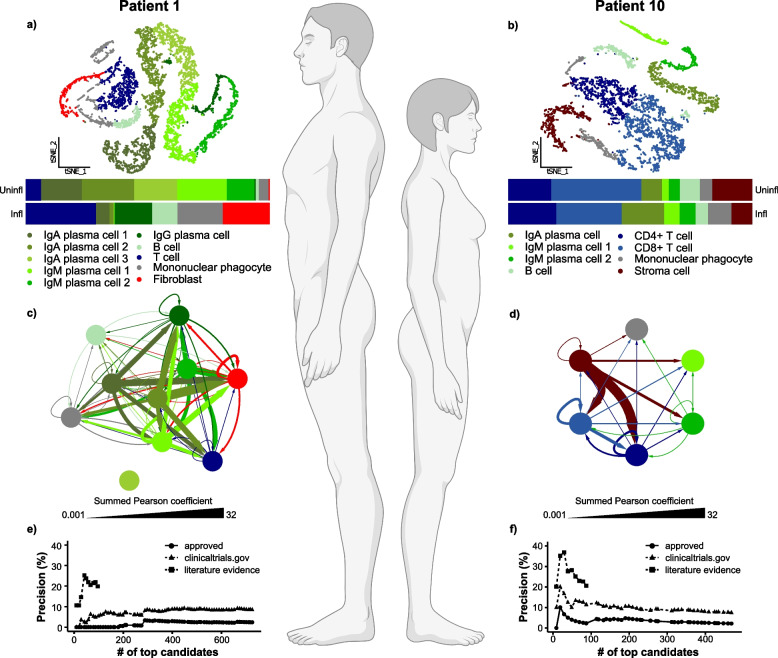
Different cellular and molecular constellations in two patients with the same diagnosis, Crohn’s disease (CD), figure from [37]. **a**–**b** scRNA-seq of intestinal biopsies showed that cell type proportions differed between two patients with CD; **c**–**d** multicellular disease models (MCDMs) of disease-associated cell types showed significant differences between the two patients. The MCDMs were constructed by first identifying upstream regulators (URs) of differentially expressed genes (DEGs) in any cell type. If such an UR was found in one cell type, a molecular interaction was inferred between that cell type and any cell type harboring the DEGs; **e**–**f** computational ranking of drugs that targeted the MCDMs showed that precision for approved CD drugs among top ranking drug candidates was high for patient 10 but not for patient 1. This prediction agreed with patient 10 responding to approved drugs, but not patient 1

On the scale of individuals, detailed characterization of health and disease mechanisms can be achieved using different types of genome-wide analyses (“multi-omics”) down to the level of single cells (Fig. 2) [37].

The latter is important because analyses of the transcriptomes of thousands of cells give sufficient statistical power to characterize disease-associated changes in an individual patient by comparing sick and healthy tissues. As shown in Fig. 2, such changes can vary greatly between two patients with the same diagnosis who will therefore require different treatments.

**Fig. 3:**
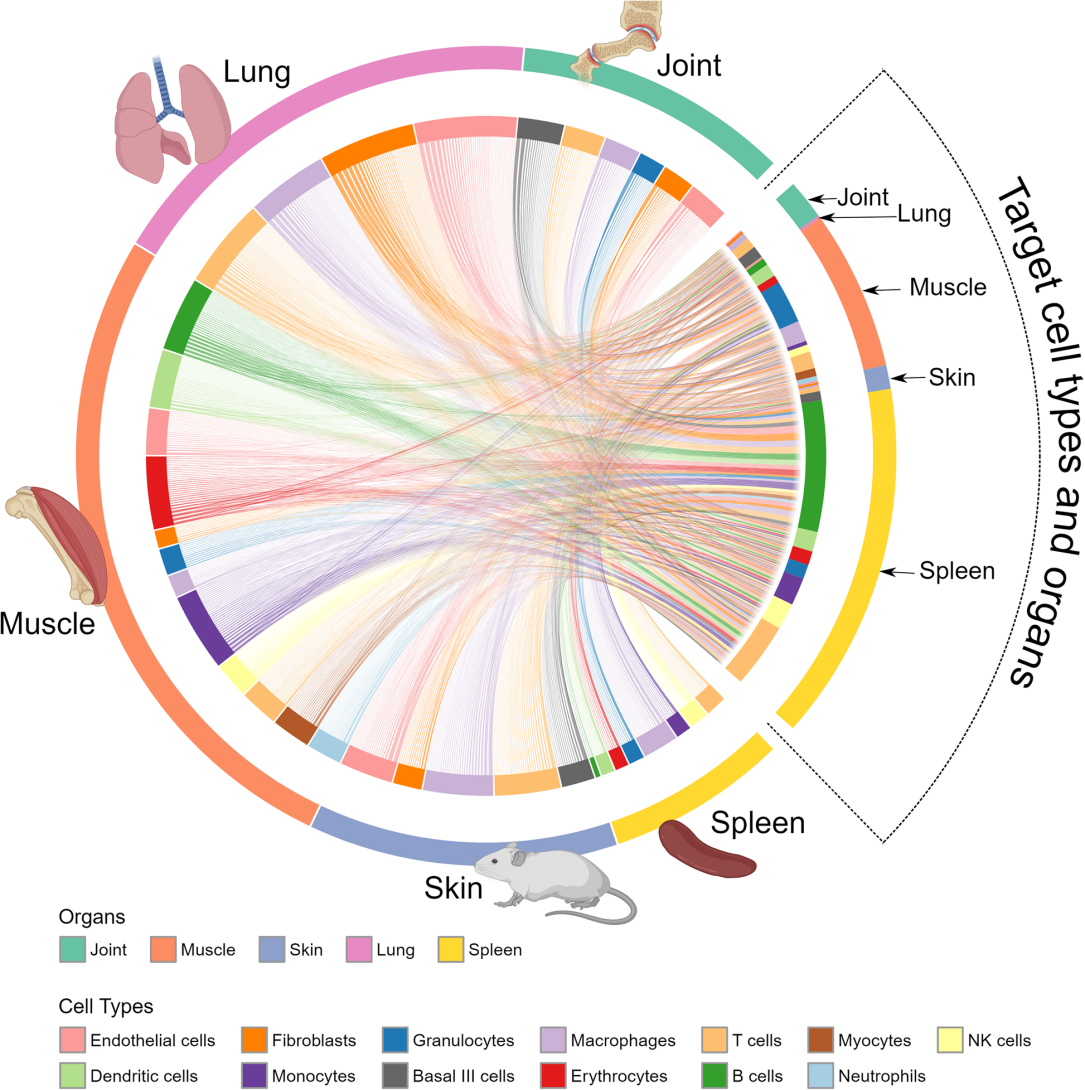
Multi-organ scRNA-seq analyses of a mouse model of arthritis showed involvement of multiple interconnected organs, not simply joints, figure from [1]. The outer circle denotes the analyzed organ, while the inner circle denotes cell types. Lines within the inner circle denote predicted molecular interactions between cell types in each organ. Those predictions were derived from bioinformatically inferring upstream regulators of differentially expressed genes in different cell types, as described in Fig. 2 [1]

Treatment of disease-associated changes is further complicated by the involvement of multiple organs with variable mechanisms in the same patients [1, 65]. A recent single-cell RNA sequencing (scRNA-seq) study of a mouse model of arthritis showed involvement of multiple interconnected organs, although only joints showed signs of disease (Fig. 3).

This heterogeneity has important clinical implications: a drug target in one organ may variably interact with the same or other genes in the same and other organs. Such variations are not possible to measure in individual patients with current diagnostic methods. This may be one explanation for why medication is ineffective in many patients.

## 2. Systems-level principles that organize health and disease mechanisms into an overarching DT structure for populations and individuals

The complexity and heterogeneity of diseases calls for systems levels to organize disease-associated changes into DTs on scales ranging from populations to individuals.

We propose that analyses of data on population-wide scales, such as those shown in Fig. 1, can potentially be developed to construct DTs of health and disease processes in whole populations (henceforth referred to as pop-DTs). Since the data and methods to construct pop-DTs have yet to be developed and identified, an exact definition of a pop-DT remains to be developed. However, a prototypic definition could be virtual representations of healthy and sick processes in populations across life spans, as well as their environmental and genetic associations. The pop-DTs should be continuously updated with relevant data from any relevant data source, such as electronic medical records, quality registries, and environmental and genetic databases. The pop-DTs should facilitate analyses to identify factors that influence health and disease to promote health and predict and prevent diseases. Construction and analyses of pop-DTs will involve huge challenges, including finding relevant data and developing methods to analyze such data. Pop-DTs should ideally describe combinations of environmental and genetic causes of health or disease. The underlying data are increasingly available in longitudinal electronic medical records, quality registries, and genome-wide databases. Pop-DTs should be continuously updated based on information from the literature and the evolution of different databases. The example in Fig. 1 may represent an early attempt to address such challenges. The result can be seen as a prototypic pop-DT. This version of pop-DT, based on natural language processing-inspired word embedding, we computed a 20-dimensional continuous “disease space,” where diseases, such as lung cancer or depression, are represented as 20-dimensional vectors. In this embedding, similar-etiology diseases tend to occur in close neighborhoods of each other.

**Fig. 4:**
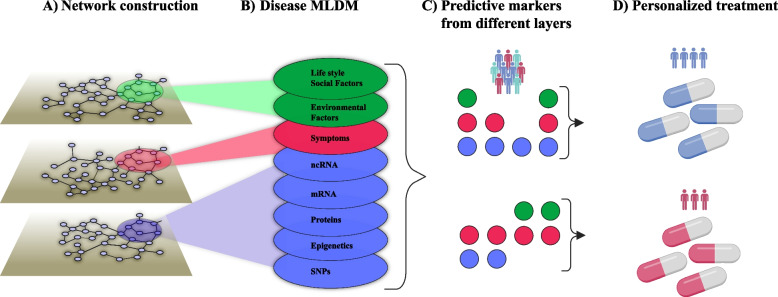
Multi-layer networks to integrate diverse disease-associated variables for personalized treatment. **A** All factors that influence a disease can potentially be described by networks. For instance, symptoms and signs that frequently tend to co-occur can be grouped into a module that represents a disease (pink oval). That disease module may be associated with corresponding modular changes in proteins (blue oval). Similarly, the disease module may be connected to co-occurring environmental factors (green oval). **B** The modules from **A** can be further subdivided into distinct sub-layers, from which **C** predictive combinations of multimodal variables from the different sub-layers can be identified based on machine learning (ML). For example, a red variable from the symptom’s module and a blue variable from any sub-layer of the molecular module. **D** Differences between such combinations can be used to personalize treatment. MLDM, multilayer disease module; nc-RNA, noncoding ribonucleic acid; PPI, protein–protein interaction; SNPs, single-nucleotide polymorphisms

Indi-DTs translate the same principles to individual patients, but at a greater resolution. Disease-associated changes can be described on multi-organ, -cellulome, and genome-wide scales, as shown in Figs. 2 and 3. The clinical importance of cellular and molecular resolution lies in that this is needed to find biomarkers and drug targets for predictive and preventive treatments. The figures also illustrate how different types of variables can be organized into networks on different scales. For example, in Fig. 2 disease-associated cell types from individual patients are connected into networks using predicted molecular interactions between those cell types. Those interactions were predicted by bioinformatically inferring the upstream regulator (UR) genes of differentially expressed genes (DEGs) in any cell type. If an UR was found in one cell type and its DEGs in another, the two cell types were connected by an edge. Importantly, networks may provide a systems-level solution that organizes multiple types of variables in a complex system and shows how they interact within and between different levels of that system, as well as with variables in other complex systems. For example, symptoms and signs of human diseases can be connected to a network. In such a network, co-occurring symptoms and signs of the same disease are interconnected into modules (like pain in the chest and left arm in myocardial infarction). Such modules can, in turn, be connected to underlying cellular and molecular networks. Similarly, networks of environmental factors can be constructed and connected into multi-layer networks that describe diseases in scales ranging from populations to individuals, as well as how they change over time (Fig. 4A and B).

Such multi-layer networks may be used to analyze the multiple relationships each node within the network has with every other node. For example, environmental effects can be depicted by recognizing the post-translational modifications of proteins in the protein–protein interaction network and their functional consequences. Ideally, tracing such relationships could lead to identification of subnetworks or modules in which the major determinants of every specific disease exist. If so, this could lead to the identification of potential drug targets that can be used to guide therapeutic strategies and drug development, including drug repurposing [66, 67]. Moreover, multi-layer networks can provide a framework from which highly predictive combinations of variables for different purposes, such as personalized treatment, can be inferred with deep learning/artificial intelligence (AI) techniques (Fig. 4C and D). These principles will be applied in a recent initiative, The Virtual Child, which aims to construct computational models of individual children’s cancer development to predict, prevent, or treat such developments, based on multi-layer networks [68]. This initiative is based on a multidisciplinary team (professional social network) consisting of patient advocates, industry partners, and basic and clinical researchers from three continents. Thus, the application of network tools to construct multi-layer networks may provide a solution to the challenge of constructing and analyzing pop- and indi-DTs. Many approaches for constructing medical DTs have been proposed and extensively reviewed elsewhere [3]. These strategies encompass advanced machine learning (ML) algorithms and computational modeling techniques, such as multi-scale models that integrate molecular, multicellular, and organismal scales, all of which are fundamental to this process. These modeling approaches may involve systems of ordinary differential equations, agent-based models, and other dynamical systems models. The latter are crucial for modeling molecular interactions within cancer cells that ultimately influence cellular phenotypes. Moreover, ML algorithms significantly contribute by identifying complex patterns and associations within large datasets, improving the efficiency and accuracy of predictions related to tumor behavior and treatments outcomes.

In the next section, we will discuss how networks can be systematically analyzed to prioritize mechanisms for predictive, preventive, and personalized medicine.

## 3. Prioritization of mechanisms, from which diagnostic biomarkers, preventive measures, or therapeutic targets can be inferred

Prioritization of disease-relevant environmental, phenotypic, and molecular changes on dynamic population-, organome-, cellulome-, and genome-wide scales are unresolved challenges.

However, recent studies point to potential solutions:

1. On the scale of pop-DTs, analyses of longitudinal data from electronic medical records or biobanks can identify the evolution of disease constellations such that the initiating mechanisms of (preclinical) diseases can be identified (Fig. 1). Combined analyses of molecular data can be used to infer early mechanisms, as well as biomarkers and drug targets for prediction and prevention.
2. On the scale of indi-DTs, the potential of single-cell-based methods for personalized medicine was recognized at an early stage [69]. Recently, several methods have been described to infer relations to clinical traits such as survival and treatment responses. These methods have been applied to multiple diseases, including cancer, and cardiological and neurological diseases [70–72]. As an example, the scGWAS (scRNA-seq assisted genome-wide association studies analysis) method was developed to investigate transcriptional changes associated with genetic variants in specific cell types and their relationship to traits in multiple complex diseases [73]. Another application is to infer drug sensitivity based on scRNA-seq data. In cancer or cardiac cells, drugs or drug combinations can be inferred by integrating analyses of single-cell expression profiles with pharmacogenomic databases [71, 72, 74, 75]. A recent study proposes a novel computational method to identify phenotype-associated cellular states that could be used to infer biomarkers to predict response to therapy and survival in order to improve prognosis and treatment [72]. Frameworks like scDrugPrio construct network models of diseases based on scRNA-seq data to prioritize drug candidates. This approach considers cell type-specific gene expression changes.
3. Dynamic multicellular disease models (MCDMs) can be analyzed to find early URs, which may be both diagnostic and therapeutic targets that predict and prevent disease in cancer, cardiological, and neurological diseases
4. Network analyses, such as centrality measures, can be used to prioritize the most central cell types in MCDMs and their modules. Those modules may be computationally matched with thousands of drugs to find the optimal ones for individual patients (Fig. 2e–f). This approach has been validated by extensive in vitro and in vivo studies [37] and is ready for clinical trials.
5. Machine and transfer learning can be used to project data about genome-wide drug responses from public databases to individual patients [76, 77].

## 4. Solutions to connect 1–3 so that medical DTs can learn from each other and emerging DTs from other fields over time

Pop- and indi-DTs are envisioned to learn and adapt continuously, providing predictive, preventive, and personalized treatment based on diverse data, as described above. The potential of linking medical DTs to emerging DTs in related fields, such as climatology, environmental pollution, and socioeconomics, was recently discussed at a series of seminars organized by the US National Academies of Sciences, Engineering, and Medicine [56, 78]. Algorithmic advances in AI [79] that can contribute to improving and integrating DTs include self-supervised learning [80–83], geometric deep learning [84–86], and generative pre-training of foundation medical models [87–92]. Collectively, these AI approaches are transforming adjacent areas, including healthcare decision support systems, and can be directly adapted to enhance the predictive power and scalability of digital twins due to their unique capabilities in handling complex, multi-modal, and data-limited environments, which are characteristic of biomedical systems across scales.

Self-supervised learning is a form of ML in which the system learns to predict part of its input from other parts of its input using a large amount of unlabeled data. In healthcare DTs, obtaining large-scale labeled datasets is often challenging due to privacy concerns, cost, and the complexity of clinical annotations [93, 94]. Self-supervised learning allows models to leverage vast amounts of unlabeled medical data (e.g., clinical notes, imaging data) to pre-train models that can be fine-tuned with minimal supervision. This is crucial for DTs, which must integrate various forms of patient data and operate in data-constrained environments. For example, a DT could learn patterns from medical images, electronic health records, or genetic data to predict missing patient records or infer future clinical events, enhancing the DT’s ability to simulate potential disease progressions even when labeled data is limited. Geometric deep learning is a recent paradigm in that generalizes deep neural network models to non-Euclidean domains such as graphs and manifolds [95]. Biological systems naturally reside on graph-structured data, such as molecular structures, protein–protein interaction networks, molecular pathways, and patient similarity networks. Geometric deep learning excels at learning from data structured as graphs, meshes, or manifolds, making it ideal for capturing the relationships and dynamics within these systems [96, 95, 97–99]. For example, in a DT of the human heart, geometric deep learning approaches could model geometries of different anatomical structures (e.g., blood vessels, muscle tissues) to simulate cardiovascular functions under different conditions. These approaches are particularly powerful in modeling spatial relationships in imaging data, which can be useful for simulating personalized disease models [100, 101].

DTs need to integrate multiple data types, such as clinical data, genomics, and imaging. Generative pre-training on large-scale medical datasets is a technique to build foundation models [87, 88, 92, 102–105] that learn medical knowledge across these modalities and can be fine-tuned for specific DT applications. Instead of training many task-specific models, we can adapt a single, generative, pre-trained model to many tasks via few-shot prompting [106–109] or fine-tuning [110–112]. For example, in virtual cell simulators [113], this approach can generate and test hypotheses in virtual environments, enabling scientists to explore scenarios and conditions that are difficult to replicate in a physical laboratory [114]. In clinical DTs, this approach could simulate patient-specific outcomes by generating treatment responses or disease progressions based on the individual’s data.

## 5. Solutions to make DTs explainable to individuals, care givers, and scientists

Integrating DTs and AI models into clinical settings presents an important challenge in ensuring these technologies are interpretable and transparent to individuals, care givers, and medical researchers. This is essential for participatory medicine, where joint decision-making between patients and health professionals is based on a clear and informed understanding of health and disease management [49]. Machine learning models often function as black boxes, making it difficult for end-users, such as clinicians and patients, to understand how predictions are made [115]. Ensuring these models are explainable without sacrificing accuracy is crucial for trust and usability in DTs [116, 117]. Explainability techniques allow us to create DT models that are more transparent. These techniques include tools that visualize how data points are connected and influence one another within the model and algorithms that break down complex predictions into simpler, more comprehensible components. One of the most effective explainability tools in DTs is attribution maps [118–120]. These maps visually represent which parts of the model’s structure—such as nodes or edges in a graph, pixels in an image, or time points in sequential datasets—contribute most to a prediction. For example, in a medical DT simulating a patient’s disease progression, attribution maps can highlight which clinical symptoms, genetic markers, or other factors are most influential in diagnosing a condition or predicting a treatment outcome [102, 121]. This visualization helps clinicians validate the model’s reasoning and makes it easier for patients to understand why certain medical decisions are recommended.

Another explainability technique involves local explainers—tools that focus on explaining individual predictions rather than the overall model behavior [122–124]. In healthcare DTs, where personalized care is essential, local explainers can offer detailed insights into why a model recommended a specific treatment or diagnosis for a particular patient. For instance, in a DT built from scRNA-seq data, local explainers can help determine why a certain cell type or gene expression pattern was critical for prediction [125]. This fine-grained understanding is especially useful in precision medicine, where individual-level explanations are often more actionable than global trends. In scRNA-seq DTs, explainable AI can be employed to trace the molecular basis of a prediction. For example, a visible neural network [126]—designed to be inherently interpretable—can illustrate which gene expressions or pathways influenced the model’s classification of cell states in a patient’s immune response [127]. This type of transparency is critical in complex systems where biological pathways are intricate, and predictions must be rooted in identifiable molecular changes. In healthcare DTs, a visible neural network could be deployed to predict hospital readmissions. Each layer of the model is structured to offer insights into why certain factors (e.g., age, comorbidities, medication adherence) influence the likelihood of readmission. By making these decisions transparent, hospitals can better allocate resources and tailor interventions for at-risk patients [128, 129].

Designing explainable interfaces for DTs tailored to patients’ preferences and educational backgrounds can enhance DTs. This might involve creating visualizations that patients can understand while providing clinicians with detailed insights [130]. For example, a DT interface that simulates the impact of lifestyle changes on disease progression could incorporate interactive elements to engage patients more effectively, adapting the presentation based on their health literacy [131]. Leveraging explainability techniques like attribution maps, local explainers, and visible neural networks can enhance the usability of DTs and foster interaction between DTs and human users [132, 133].

## 6. Solutions to disseminate DTs on a global scale for equitable and effective health in accordance with the 2030 agenda for sustainable development

As evidenced by the Virtual Child Project, which spans three continents, many of the computational solutions underlying DTs are independent of geographical location. This supports that DTs may contribute to improved and equitable health on a global scale based on collaborative efforts between developing and developed countries. There are several successful examples of such collaborations, aiming at global health digitalization, including concrete examples such as an automated pipeline for virtual drug discovery and clinical applications such as digital or AI-supported diagnostic protocols in low-resource settings [134–136].

## 7. Solutions to address social, psychological, organizational, ethical, regulatory, and financial challenges and opportunities

Clinical implementation of DTs will involve a wide range of challenges. As recently discussed, many of these challenges are generic for implementation of computational science in different fields [56]. One important example is gender differences in how digital technologies and health care are perceived, used, and led in different countries [137, 138]. Such differences can be disadvantageous for women—especially women of racial or ethnic minority backgrounds. Another question can be data ownership: can a patient be asked to share increasingly detailed information from her DT as a resource for clinicians treating patients with similar characteristics, or for use in clinical or industrial research, such as drug discovery? Addressing this question requires integrated solutions to tackle challenges in ethics, data security, and regulatory issues [139]. However, despite national differences in evaluation and approval processes, computational modeling tools for clinical purposes have entered in the market. The FDA has implemented pre-qualification programs to speed up the regulatory processes of digital tools. Additionally, protecting the privacy and rights of an individual’s DT is crucial, especially as it incorporates sensitive, multiscale data. The analyses for example federated data analysis with evolving computational approaches that protect privacy even in population-based studies. A white paper from the US National Academy of Science recently recommended that the potential of digital twins to “accelerate scientific discovery and revolutionize health care” would merit an integrated agenda to harmonize research across sectors and focus efforts on realistic applications. These efforts should be “crosscutting” to help “advance the mathematical, statistical, and computational foundations underlying digital twin technologies.” However, the white paper also stated that there is a “lack of adopted standards in data generation” that “hinders the interoperability of data required for digital twins.” Finally, the reports urged “fundamental challenges include aggregating uncertainty across different data modalities and scales as well as addressing missing data” [140].

## Concluding remarks

While implementation of DTs for predictive, preventive, and personalized medicine will involve huge and diverse challenges, these must be balanced against the suffering and costs resulting from the many patients for whom today’s diagnostics and therapeutics are ineffective.

These challenges arise from the intricate nature of diseases, which involve complex interactions among thousands of genes across various cell types and organs. Disease progression can vary significantly between patients and overtime, influenced by a combination of genetic and environmental factors. DTs are increasingly recognized as a potential solution to address these challenges in healthcare. Early clinical applications of DTs have already emerged for endocrine, cardiological, and malignant diseases, as well as for hospital workflow optimization.

These applications demonstrate the versatility and potential impact of DT technology in healthcare. However, widespread implementation of DTs in healthcare faces several challenges:

1. Biological complexity: characterizing dynamic molecular changes across multiple biological scales.
2. Data integration: developing computational methods to integrate diverse data types into digital twins.
3. Prioritization: identifying and prioritizing disease mechanisms and therapeutic targets.
4. Interoperability: creating digital twin systems that can learn from and interact with each other.
5. User interface: designing intuitive interfaces for patients and clinicians.
6. Global scaling: expanding digital twin technology globally to ensure equitable healthcare access.
7. Ethical and regulatory considerations: addressing ethical, regulatory, and financial aspects of digital twin implementation.

Addressing these challenges as proposed by the 2030 agenda will require global collaborations between developed and developing countries, as well as patient organizations, health care professionals, academic and industrial researchers, politicians, and regulatory bodies.

This could pave the way for a more predictive, preventive, and personalized approach to medicine. The successful implementation of digital twins has the potential to transform healthcare delivery and significantly improve patient outcomes. As research progresses and technology advances, digital twins may become an integral part of healthcare systems worldwide, offering tailored solutions for individual patients and enhancing overall healthcare efficiency.

## Data Availability

No datasets were generated or analyzed during the current study.

## Abbreviations

DT: Digital twin
WBM: Whole-body models
NSF: National Science Foundation
US: United States
CD: Crohn’s disease
MCDM: Multicellular disease model
UR: Upstream regulator
DEG: Differentially expressed gene
ML: Machine learning
MLDM: Multilayer disease module
nc-RNA: Noncoding ribonucleic acid
PPI: Protein-protein interaction
SNPs: Single-nucleotide polymorphism
AI: Artificial intelligence
scGWAS: ScRNA-seq assisted genome-wide association studies analysis
